# Ensemble Transfer Learning for Fetal Head Analysis: From Segmentation to Gestational Age and Weight Prediction

**DOI:** 10.3390/diagnostics12092229

**Published:** 2022-09-15

**Authors:** Mahmood Alzubaidi, Marco Agus, Uzair Shah, Michel Makhlouf, Khalid Alyafei, Mowafa Househ

**Affiliations:** 1 College of Science and Engineering, Hamad Bin Khalifa University, Doha P.O. Box 34110, Qatar; 2 Sidra Medical and Research Center, Sidra Medicine, Doha P.O. Box 26999, Qatar

**Keywords:** image segmentation, ensemble transfer learning, fetal head, gestational age, estimated fetal weight, ultrasound

## Abstract

Ultrasound is one of the most commonly used imaging methodologies in obstetrics to monitor the growth of a fetus during the gestation period. Specifically, ultrasound images are routinely utilized to gather fetal information, including body measurements, anatomy structure, fetal movements, and pregnancy complications. Recent developments in artificial intelligence and computer vision provide new methods for the automated analysis of medical images in many domains, including ultrasound images. We present a full end-to-end framework for segmenting, measuring, and estimating fetal gestational age and weight based on two-dimensional ultrasound images of the fetal head. Our segmentation framework is based on the following components: (i) eight segmentation architectures (UNet, UNet Plus, Attention UNet, UNet 3+, TransUNet, FPN, LinkNet, and Deeplabv3) were fine-tuned using lightweight network EffientNetB0, and (ii) a weighted voting method for building an optimized ensemble transfer learning model (ETLM). On top of that, ETLM was used to segment the fetal head and to perform analytic and accurate measurements of circumference and seven other values of the fetal head, which we incorporated into a multiple regression model for predicting the week of gestational age and the estimated fetal weight (EFW). We finally validated the regression model by comparing our result with expert physician and longitudinal references. We evaluated the performance of our framework on the public domain dataset HC18: we obtained 98.53% mean intersection over union (mIoU) as the segmentation accuracy, overcoming the state-of-the-art methods; as measurement accuracy, we obtained a 1.87 mm mean absolute difference (MAD). Finally we obtained a 0.03% mean square error (MSE) in predicting the week of gestational age and 0.05% MSE in predicting EFW.

## 1. Introduction

Ultrasonic imaging, also known as ultrasound, is frequently utilized in clinical assessment since it does not include ionizing radiation, and it is less expensive than computed tomography (CT) and magnetic resonance imaging (MRI) [1]. Women usually have one to three ultrasounds during pregnancy. If the lady is pregnant with twins or is at high risk, ultrasounds may be required more frequently [2]. Ultrasound may be utilized in various prenatal diagnostic situations, including: confirming the pregnancy and the position of the fetus, calculating the gestational age of the fetal baby, verifying the number of fetal bodies, examining fetal development, examining the amounts of the placenta and amniotic fluid, identifying congenital disabilities, looking into complications, and other prenatal tests [3]. When ultrasound is routinely used in early pregnancy, it will result in an earlier detection of problems and an improved management of pregnancy complications, which is better than relying on clinical indicators such as bleeding in early pregnancy [4]. Halle et al. [5] reported that 1111 women received prenatal treatment at primary care health centers in their health cohort. Ninety-five percent of women reported having some fetal ultrasound scan prior to the 19th week scan, and 64% reported having two or more scans during this period. Seventy-eight percent of women decided to participate in week 11–14 screening for fetal abnormalities. Therefore, ultrasound is the preferable option for prenatal care compared to other imaging modalities, because it allows for the recognition and measurement of anatomical structures that can be used as guidelines for physician assessment of the fetal health status [3].

**Figure 1:**
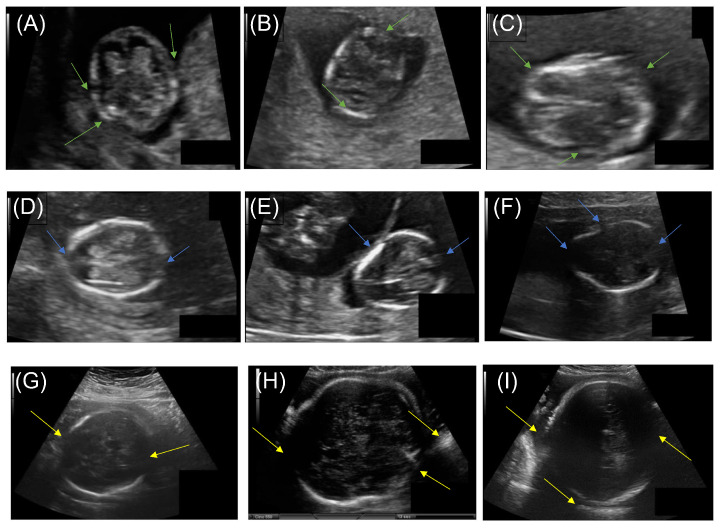
Typical prenatal ultrasound images from each trimester. (**A**–**C**) First trimester, green arrows indicate blurred fetal head and artifacts. (**D**–**F**) Second trimester, blue arrows indicate poor signal-to-noise ratio and reflection from the fetal membranes and amniotic fluid interface. (**G**–**I**) Third trimester, yellow arrows indicate speckle noise and standard sutures or ultrasonography artifacts.

Many clinical ultrasonography diagnostics necessitate the use of anatomical structure measurements that are clear and reliable. These measurements are used to estimate fetal gestational age and weight, which is essential for monitoring growth patterns during pregnancy [6]. Abdominal circumference (AC), femur length (FL), crown–rump length (CRL), occipitofrontal diameter (OFD), biparietal diameter (BPD), and head circumference (HC) are some of the biological characteristics that may be measured during a prenatal checkup [7]. In the 13th to 25th week of pregnancy, obstetricians and gynecologists may calculate the fetus’s gestational age and weight, evaluate the fetus’s growth, and decide if aberrant head development is suspected, by measuring the fetus’s HC [8]. When measuring HC in clinical practice, the procedure is performed manually by either overlaying an ellipse on the fetal skull or by recognizing landmarks that delimit the central head axis. Despite this practice, the manual delineation raises concerns about measurement repeatability and time consumption, since ultrasound imaging is prone to various errors, including motion blurring, missing borders, acoustic shadows, speckle noise, and a low signal-to-noise ratio [9]. As a result, interpreting ultrasound images becomes extremely difficult, necessitating the use of skilled operators. Figure 1 shows ultrasound image samples that are noisy and indistinct, with an incomplete head contour; additionally, the fetal skull is not evident enough to be detected in the first trimester, as indicated in the samples obtained from the public dataset [10].

Traditional approaches for fetal biometric segmentation and measures have been under investigation for the past decade. As a result of the development of these approaches, workflow efficiency has been increased by lowering the number of steps required for routine fetal measures and examination time [6]. The randomized Hough transform [11], semi-supervised patch-based graphs [12], multilevel thresholding circular shortest paths [13], boundary fragment models [14], haar-like features [7], active contouring [15], morphological operators [16], the difference of Gaussians [17], and deformable models [18] have all been used in previous HC measurement studies.

With the advancement of deep learning technology in recent years, integrating medical images and artificial intelligence has emerged as a popular study area in medicine [19]. Convolutional neural networks (CNNs) have rapidly gained popularity as a powerful tool for many image processing applications, including classification, object identification, segmentation, and registration, among others [20]. As a result, the field of medical image segmentation is exploding with new applications. A few representative designs of CNNs are fully convolutional networks (FCNs) [21], UNet [22], and three-dimensional VNet [23].

### 1.1. Contributions

Numerous challenges remain for prior traditional and deep learning methods, including segmenting regions with missing edges, the absence of textural contrast, the specification of a region of interest (ROI), and background detection. These difficulties can be overcome using ensemble learning. Nowadays, CNNs are evolving towards lightweight architectures that can be integrated in edge computing frameworks [24], but prior mentioned techniques required a lengthy training period, high network parameters, high image resolution, and costly resources to run a heavy model. However, these issues may be mitigated by fine-tuning a pre-trained lightweight network. Finally, earlier studies did not explore the feasibility of utilizing machine learning and segmented image measurements to determine fetal gestational age (GA), estimated fetal weight (EFW), and abnormality signs. In this regard, this work proposes a complete pipeline for automatic segmentation and measuring the fetal head in two-dimensional (2D) ultrasound images, followed by a prediction of the fetal gestation age and weight. Below is a summary of technical contributions:

1. We fine-tuned eight segmentation networks using a pre-trained lightweight network (EffientNetB0) and employed weighted voting ensemble learning on the trained segmentation networks to obtain the optimal segmentation result.
2. We extensively evaluated the ensemble transfer learning model (ETLM) by performing three-level evaluations: fetal head segmentation evaluation, predicted mask and post-processing quality assessment, and head measurement evaluation.
3. We generated a new fetal head measurement dataset and manually labeled it by adding fetal gestation age and weight.
4. We trained multiple regressions model to predict fetal GA and EFW to address the limitation of the current formulas (Equations (21) and (22)).
5. We evaluated the regression model result using an expert obstetrician, and a longitudinal reference using Pearson’s correlation coefficient (Pearson’s r).

### 1.2. Organization

The following is the paper’s organization: Section 2 discusses relevant research on fetal head segmentation, HC measurement, and fetal GA and EFW calculation. Section 3.1 discusses the dataset and our methodology pipeline in depth. Section 4 contains details about the experiment and evaluation methods. Section 5 presents the results, discussion, and a comparison with state-of-the-art works. Section 6 highlights the strengths and limitations of the research. Finally, Section 7 covers a conclusion and future work.

## 2. Related Work

Our works deals with fetal head segmentation using traditional approaches and deep learning, HC measurement, and the calculation of GA and EFW. It is impossible to provide here an extensive overview of the literature related to these topics. We refer readers to the survey and review [4,25,26,27]. In the following, we discuss the methods that are most closely related to our work.

### 2.1. Fetal Head Segmentation

#### 2.1.1. Traditional Approaches

Many works have used a variety of machine learning algorithms for fetal head segmentation. One example is the probabilistic boosting tree (PBT), which has been utilized for AC measurement [28]. A random Hough transform approach developed by Lu et al. [29] has been used to recognize incomplete ellipses in images with severe noise. However, their method may fail to detect the fetal head in low-contrast ultrasound images. Zhang et al. [30] developed multi-scale and multi-directional filter banks to extract anatomical structures and texture characteristics from fetal anatomical structure and texture images. Li et al. [31] used a prior knowledge of fetal head circumference to obtain the region of interest with random forest and detect the fetal head edge with phase symmetry. They found that their method performed poorly on fitting the fetal skull from ultrasound images with partially missing features taken in late pregnancy. A complex approach, such as [10], retrieved the HC by using haar-like characteristics to train a random forest classifier to detect the fetal skull, and employed the Hough transform, dynamic programming, and elliptical fitting. Even though these previous approaches produced promising findings, they were only tested on small datasets of specific pregnancy trimesters, and fetal ultrasound images at different stages of pregnancy vary in their inherent characteristics. Therefore, aspects such as the efficiency and accuracy of current traditional methods for automatic fetal head segmentation and HC biometry performance need to be improved because with current limitations, they are not adequate for accurate and reliable diagnosis by physicians.

#### 2.1.2. Deep Learning

Deep learning techniques began to grow in popularity because of advancements in technology. This method had significantly better skills in image processing tasks due to their promising capabilities. In particular, CNN has emerged as a top choice for medical image classification, segmentation, and object detection [4]. UNet [22] is a network often used for biomedical image segmentation because of the symmetric structure observed in the images, allowing for the efficient use of skip connection layers and the reduced computing complexity. First, a feature map is extracted from an image via the encoders in the UNet architecture. Then, the decoders cascade their corresponding encoded feature maps to extract even more spatial information from the image. Several modified U-shape networks [32,33,34] been used to segment fetal ultrasound images, and have achieved notable results. The segmented images obtained can be utilized to detect the elliptic fetal skull and calculate the fetal HC. Sobhaninia et al. [32] proposed a multi-task deep network structure based on the LinkNet topology. They segmented fetal ultrasound images using LinkNet [35] capabilities. Their experimental results revealed that multi-task learning yields better segmentation outcomes than a single-task network. Qiao and Zulkernine [36] presented an expanded UNet model [22] with dilated convolution layers and Squeeze-and-Excitation (SE) blocks to enhance segmentation of the fetal skull border and skull in 2D ultrasound images. They used dilated convolution extracting features from a more extensive spatial range to detect edges without increasing the model complexity, and to measure fetal HC.

Desai et al. [37] proposed the DUNet architecture based on the UNet. The image and its scattering coefficients (SC) are inputs for the DUNet. Each of these inputs has an encoder. The encoders’ outputs are combined and sent into a single decoder, eliminating data augmentation and reducing the training time. Aji et al. [38] utilized UNet with pixel-wise classification to increase ROI image classification performance. Each pixel is divided into four classes: maternal networks have horizontal direction patterns, higher head borders have concave arc patterns, lower head boundaries have convex arc patterns, and the rest. The LinkNet network [35] was used as inspiration for the multi-scale and low-complexity structure of the proposed network by Sobhaninia et al. [39]. They were able to lower the number of convolutional layers in mini-LinkNet. The LinkNet network includes four encoder blocks; however, the mini-LinkNet network has just three encoder blocks, which appear to be more efficient and may retain image characteristics. These researchers demonstrate that employing a light network for the segmentation of the fetal head can lead to the intended result. Brahma et al. [40] proposed accurate binary DSCNNs for medical image segmentation. The networks’ encoder and decoder structures use parameter-free skip connections to binarize them. Asymmetric encoder–decoder DSCNNs feature pyramid networks with asymmetric decoders and spatial pyramid pooling with atrous convolutions are evaluated on the fetal head image. An intensely supervised attention-gated (DAG) VNet method was introduced by Zeng et al. [41] for automated two-dimensional ultrasound image segmentation of the fetal head. Attention gates (AGs) and deep supervision were added to the original VNet architecture. Multi-scale loss functions for deep supervision were also introduced. The suggested DAG VNet technique increased segmentation accuracy while increasing the convergence speed by including the attention mechanism and deep supervision strategy. Xu et al. [42] proposed a vector self-attention layer (VSAL) and a context aggregation loss (CAL) in CNN. Geometric priors and multi-scale calibration were developed for long-range spatial reasoning. Unlike nonlocal neural networks, VSAL could concurrently attend to spatial and channel information, and VSAL consider multi-scale information by applying geometric priors and multi-scale calibration. They also introduced context aggregation loss (CAL) as an additional benefit to VSAL. CAL analyzes global contextual information and intra- and inter-class dependencies. Then, they use VSAL as the backbone to replace the convolutional layers. The suggested VSAL outperforms various mainstream methods on prenatal images. It also shows the method’s adaptability to various segmentation networks. Skeika et al. [43] presented an innovative approach for automatically segmenting a fetal head in 2D ultrasound images. The suggested approach, called VNet-c, uses the original VNET [23] but includes several modifications. The modifications include pre-processing, batch normalization, dropout use, data augmentation, loss function, and network depth adjustments. The authors in [23] evaluated the suggested method’s performance quantitatively using negative and positive rates. The fetal head and abdomen segmentation in an ultrasound image was performed by Wu et al. [44] using a cascaded FCN in combination with context information. Sinclair et al. [45] used an VGG-16 FCN to segment the fetal head in ultrasound images taken during the second trimester. Object detection is also used with fetal ultrasound images, using fast regions convolutional neural networks (R-CNN) and FCN. Al Bander et al. [46] developed a method to identify the fetal head boundary using a combination of fast R-CNN and FCN that included target localization and segmentation.

All of the works mentioned above did not consider the resource constraints and training time. To the best of our knowledge, this is the first trial to employ ensemble transfer learning for fetal head segmentation and to develop a lightweight model with low resources and less training time with respect to model accuracy.

### 2.2. Fetal Head Measurement

Various methods have been proposed to derive accurate geometric measurements from segmentation masks, such as the head circumference and radii. In general, most methods consider various elliptical models for representing the fetal head shape. Zhang et al. [47] proposed a method that estimates the HC from ultrasound images without segmentation. Their technique uses a regression CNN, for which they tested four networks of varying complexity and three regression losses. It is the first direct measurement of fetal head circumference without segmentation. Region-proposal CNN for head localization and centering, and regression CNN for precise HC delineation are proposed by Fiorentino et al. [48]. Then, distance fields are used to train the regression CNN. In order to make the network task of directly regressing the HC line easier, a distance field is used to smooth the HC line. Skeika et al. [43] used their own designed algorithm to calculate HC from the predicted mask. Zeng et al. [41] used fitted ellipses to calculate HC biometric measurements based on the following formula:

(1) $$HC=2\pi \times SemiAxis\;b+\left(4\times \left(SemiAxis\;a-SemiAxis\;b\right)\right)\;$$

where SemiAxisa and SemiAxisb are the major and minor axes of the ellipse. Qiao and Zulkernine [36], and Li et al. [49] used the direct least square fitting of ellipses to measure the HC. A second-order polynomial, such as the following, can be used to express a generic conic:

(2) $$\begin{array}{c} F\left(\vec{a},\vec{x}\right)=\vec{a}\cdot \vec{x}=ax^{2}+bxy+cy^{2}+dx+ey+f=0 \end{array}$$

where $\;\vec{a}={\left[a,b,c,d,e,f\right]}^{T}$ and $\;\vec{x}={\left[x^{2},xy,y^{2},x,y,1\right]}^{T}$ Aji et al. [38] used an ellipse fitting method comparable to the ElliFit method, in which the median value of the largest area’s edge points is generally sought. Following these two operations, five ellipse parameters are acquired and used for elliptical representation. Once these two numbers have been calculated, they are multiplied by the pixel size of the input image. After obtaining the parameters, it is possible to approximate HC by computing the ellipse border using the following formula:

(3) $$HC=0.5\times \pi \times \left(a+b\right)$$

where *a* and *b* are the major and minor axes of the ellipse.

In this work, we propose a geometry fitting framework for computing fetal head measurements, composed of the following processing steps: smoothing, parameterization, resampling, the linear least square minimization process for fitting an explicit model, and the accurate geometric distance between points. The model is parameterized in a way that the Jacobian and the geometric parameters of the best-fit ellipse can be computed in closed-form.

### 2.3. GA and EFW Calculation

In general, the starting day of the last menstrual period (LMP) is used to calculate gestational age (GA). However, in around 40% of pregnancies, the LMP is unknown or unreliable [50]. Ultrasound provides more reliable information on GA and is primarily acknowledged as the preferred approach. Ultrasound can determine GA more accurately than physical examination in most pregnancies. During the first trimester, the gestational sac mean diameter and crown–rump length (CRL) are used to determine GA. Measurements of the fetal head, torso, and extremities are most frequently used in the second and third trimesters. A combination of BPD, HC, abdominal circumference (AC), and femur length (FL) are typically measured parameters that are used to calculate the GA [51]. Many other variables have been examined and linked to GA, but few increase the accuracy of GA estimation [52].

In fetal medicine, the ultrasound estimation of fetal weight (EFW) is essential for prenatal care. EFW helps the physician to determine whether fetuses are the proper size for their gestational age (GA), small (SGA), or large (LGA) [53]. The EFW is calculated from the HC, BPD, FL, and AC measurements. The formulas of Hadlock et al. [54] were the most accurate, with the lowest Euclidean distance and the highest absolute mean error being less than 10%. Hadlock et al. [54] (Equation (4)) used HC, AC, and FL measurements with or without BPD. They found a robust connection between birth weight and EFW based on HC, AC, and FL measurements [55].

(4) $$log_{e}\left(EFW\right)=1.326-0.00326\times AC\times FL+0.0107\times HC+0.0438\times AC+0.158\times FL.$$

where AC, FL, and HC are the measurements that are mentioned in the previous paragraph. To the best of our knowledge, this is the first trial study to employ a machine learning regression model to predict fetal GA and EFW based on the fetal head, without the need for other measures such as AC and FL.

## 3. Materials and Methods

### 3.1. Methodology

**Figure 2:**
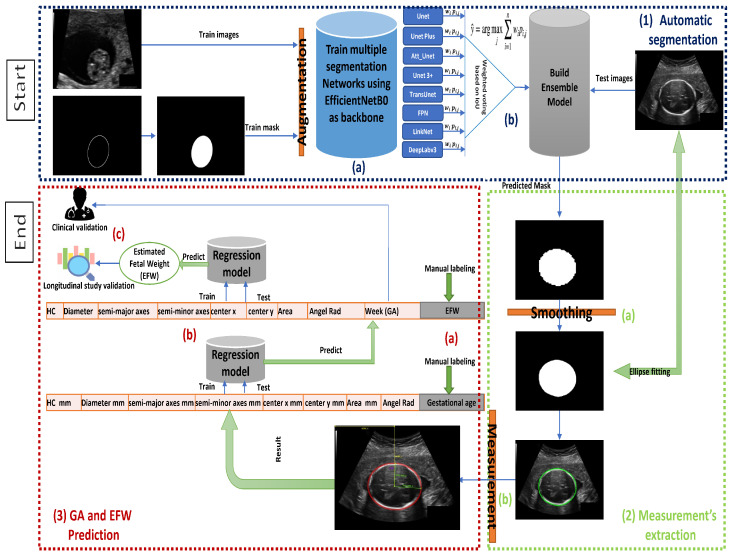
Workflow of the pipeline followed in this paper: Block (1) for fetal head segmentation. Block (2) for smoothing and measuring. Block (3) for fetal GA and weight prediction red.

Figure 2 illustrates the workflow of a full end-to-end pipeline that was proposed to achieve the main contribution of this paper. The pipeline components are demonstrated in three main blocks, as seen in Figure 2. These blocks can be subdivided as follows:

1. Automatic segmentation: takes as an input a ultrasound image, and gives an output binary mask representing the fetal head.
  (a) Eight segmentation models are fine-tuned independently using the pretrained CNN EfficientNetB0 as the feature extractor.
  (b) The segmentation predictions of these models are integrated through ETLM.
2. Measurements extraction: from an automatically computed and smoothed binary mask, we fit an analytic explicit ellipse model that we use for computing the geometric measurements of interest, such as semi-axis and head orientation.
  (a) Image post-processing and smoothing.
  (b) Fetal head measurement.
3. GA and EFW Prediction: from measurements and manual annotations, we fit a regression model that is able to predict GA and EFW, which we validate clinically.
  (a) Generate new GA and EFW dataset and labeling.
  (b) Trained multiple regression models on the new dataset.
  (c) Clinical and longitudinal study validation.

In the following, we firstly describe the dataset used in this study and detail the various components of the framework.

### 3.2. Dataset

The dataset on which the suggested approach was evaluated is available on Grand Challenge HC18 (https://hc18.grand-challenge.org/, accessed on 21 May 2022). Table 1 shows the distribution of the dataset during various trimesters of pregnancy. The dataset consists of ultrasound images, a training set of 999 images, a CSV file containing the HC and pixels size of each image, a test set of 335 images, and a CSV file containing only the pixel size of each image. These images were taken from 551 women throughout their first, second, and third trimesters of pregnancy. The images were acquired from the Radboud University Medical Center’s Department of Obstetrics in Nijmegen, Netherlands, using the Voluson E8 and the Voluson 730 (General Electric, Boston) [10]. All data were collected and anonymized by qualified sonographers following the Declaration of Helsinki. The local ethics commission authorized the data collection and usage for research purposes (CMO Arnhem-Nijmegen). Each image was 800 × 540 pixels in size, with pixel sizes varying from 0.052 to 0.326 mm due to sonographer modifications to accommodate varying fetus sizes. The sonographer manually marked each image by drawing an ellipse corresponding to the skull portion. The unique issues in the images are depicted in Figure 1. The difficulties included the head being in a variable location in the image, incomplete ellipse, and the fetal head’s dimensions fluctuating over the gestational trimesters.

We augmented the dataset to increase the network’s resilience, prevent overfitting of the training data, and improve the network’s generalization ability. Nine images were generated for each image and mask in the training set using [56]. The final augmented training set includes: (1) Center Crop, (2) Random Rotate, (3) Grid Distortion, (4) Horizontal Flip, (5) Vertical Flip, (6) Random Brightness, (7) Sharpen, (8) Affine Transformation, (9) Fancy PCA, and (10) Original Image. The total number of training sets became 9990 images and 9990 masks.

### 3.3. Ensemble Transfer Learning Model (ETLM)

#### 3.3.1. Transfer Learning

Transfer learning is the capacity of a system to recognize and apply information from one area to another. Transfer learning has three levels. First, full-adaptation uses a pre-trained network’s weights and updates during training. Second, partial-adaptation starts with a pre-trained network but freezes the first few layers’ weights and updates the final layers during training. Third, zero-adaptation uses a pre-trained model to establish the weights for the whole network without updating any layers [57].

**Figure 3:**
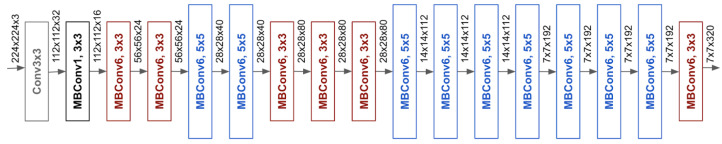
The architecture of EfficientNetB0.

This work took weights from a lightweight network (EfficientNet) and then fine-tuned them on prenatal ultrasound images. Because the dataset consists of medical images, the full-adaptation approach was used. To ensure that the best model was selected for low cost and efficiency, the lightweight EfficientNet [58] versions from B0 to B3 were utilized. EfficientNetB0 was selected based on the obtained result. EfficientNetB0 was used as the backbone (encoder) for different segmentation networks; therefore, the last block, which includes the dense layer, was removed, as seen in Figure 3.

#### 3.3.2. Ensemble Learning

Many artificial intelligence applications have significantly benefited from the use of ensemble learning, a machine-learning approach that uses numerous base learners to construct an ensemble learner for improved generalization of the learning system. A voting ensemble (sometimes known as a “majority voting ensemble”) is a type of ensemble machine learning model that incorporates predictions from several other models to arrive at a final prediction [59]. When applied effectively, it can help models perform better than any of the individual models. Voting ensembles combine the results of numerous models to arrive at a final result. For example, the predictions for each label are added together, and the label with the most votes is predicted. Almost the same results were obtained across all segmentation models in our study. Therefore, using a voting ensemble is practical when two or more models perform well on a predictive modeling task.

The models must all agree on most of their predictions for the ensemble to work. Hence, each model’s contribution is proportionate to its capacity or competence in a weighted average or weighted sum ensemble. A weighted average forecast begins by assigning each ensemble member a fixed weight coefficient [60]. A percentage of the weight may be represented as a floating-point number in the range of 0 to 1. Consider a case of three-segmentation models with three fixed weights of 0.2/0.3/0.4, where larger weights indicate a better performing model. It is possible to achieve the ideal average weight using classification accuracy or negative error, depending on the competence of each model. In this work, we used Intersection Over Union (IoU) to determine the optimal average weight for each of our eight segmentation models. The following equation is the base of weighted voting ensemble learning:

(5) $$\hat{y}=\underset{j}{arg max}\sum_{i=1}^{n}W_{i}P_{i,j}$$

where $P_{i,j}$: predicted class membership probability of the *i* classifier for class label *j* and $W_{i}$: optimal weighting parameter.

The weighted voting method was applied to eight segmentation models to find the final prediction’s optimal average weight. The segmentation models include UNet [22], UNetPlus [61], AttUNet [62], UNet 3+ [63], TransUNet [64], Feature Pyramid Network (FPN) [65], LinkNet [65], and DeepLabv3 [66]. All models were trained on the same parameter. Further, the hyperparameter tuning method [67] was applied to select a set of optimal hyperparameters, including optimizer, learning rate, loss function, and trainable parameters for the eight models, as seen in Table 2.

#### 3.3.3. Image Pre-Processing

As seen in Table 2, three image preprocessing steps were applied to eliminate undesirable distortions and to highlight certain image features. The three steps can be summarized as follows:

1. Normalization: the ultrasound image intensity range is 0 to 255. Therefore, we applied a normalization technique for shifting and rescaling values to fit in a range between 0 and 1. The Normalization Formula is as follows:
  (6) $$Z=\frac{X-X_{min}}{X_{max}-X_{min}}$$
  where *Z*: the normalized value in the image, *X*: the original value in the image, $X_{min}$: the minimum value in the image, and $X_{max}$ the maximum value in the image .
2. Resizing: The original image and mask size is 800 × 540 pixels; the images and masks were resized into two different sizes, and the difference between the two inputs, 64 × 64 and 128 × 128, is compared to evaluate the lightweight models and to use low-cost resources. In addition, while the original mask intensity was only two values, 0 and 255, after mask resizing, the intensity of the masks randomly ranged between 0 and 255. Therefore, the threshold of the resized masks had to be set to the original intensity, where 0 represents black pixels, and 255 represents white pixels. Finally, Softmax [68] was used as the output function; therefore, we had to encode the mask values to 0 for black and 1 for white pixels.
3. One-Hot encoding: One-hot encoding is not often used with numerical values (images). In this study, because the output function is Softmax and the loss function is categorical focal Jaccard loss, it is recommended that one-hot encoding be used. The class representing white pixels is (0, 1), and the class representing black pixels is (1, 0).

#### 3.3.4. Hybrid Loss Function and Optimizer

As part of the ensemble transfer learning process, selecting the appropriate loss functions increased segmentation accuracy during subsequent inference time. Therefore, various loss functions were used for medical image segmentation [69]. This work used hyperparameter tuning to comprise the best loss function based on the IoU score. The optimal loss function was the categorical focal Jaccard loss (CFJL), which is a combination of the categorical focal loss (CFL) [70] and Jaccard loss (JL) [71], as defined below:

(7) $$CFL\left(GT,PR\right)=-GT\cdot \alpha \cdot {\left(1-PR\right)}^{\gamma }\cdot log\left(PR\right)$$

(8) $$JL\left(A,B\right)=1-\frac{A\cap B}{A\cup B}$$

(9) CFJL=CFL+JL

Among the different optimizers, Adam and RMSProp [72] achieve accurate segmentation. The result demonstrates that the loss value of the Adam and RMSProp optimizers was lower than the others. However, using RMSProp with schedule learning rate and step decay that drops the learning rate (LR) by a factor every few epochs, it outperformed Adam. The step decay learning rate was defined as below:

(10) $$LR=Initial\;LR\times drop^{\left\lfloor \frac{epoch}{epochDrop}\right\rfloor }$$

### 3.4. Measurements Extraction

#### 3.4.1. Post-Processing

Multi-smoothing and edge detection techniques were applied as post-processing to correct the defective segmented mask and improve the segmentation results. The aim was to smooth and sharpen the ellipse of the contour. Among various smoothing techniques, we employed a median filter combined with morphological image processing in our scenario, where the median filter is a non-linear digital filter that suppresses pulsed (non-stationary random process) interference by eliminating all suspicious readings. The filter calculates the median output value from a set of input data (see Equation (11)) [73].

Morphological image processing is a technique that deals with the shape or morphology of picture features. Morphological operations are well suited to the processing of binary images, since they rely solely on the relative ordering of pixel values, rather than their numerical values. Greyscale images can also be subjected to morphological techniques in which the light transfer functions are unknown, and where the absolute pixel values are of no or small importance. In our scenario, a pixel is in the neighborhood if its Euclidean distance from the origin is less than the ideal value of 25 [74]. This combination of median filter and morphological process provided the best result. Figure 2 illustrates the predicted mask before and after the smoothing.

(11) $$\hat{f}\left(x,y\right)=\underset{\left(s,t\right)\in S_{xy}}{\mathrm{median}}\left\{g\left(s,t\right)\right\}$$

where g(s,t) is noise, and the median filtering method is to sort the pixels in the sliding filter window, then the output pixel value $\hat{f}\left(x,y\right)$ of the filtering result is the median value of the sequence [75].

#### 3.4.2. HC Measurements

After the post-processing stage, the predicted mask is ready for measurements, which are obtained through fitting an ellipse model to the extracted contour. The task of fitting an ellipse model on top of scatter measurements is still considered a challenging problem by the computer vision and computational geometry community [76]. In our case, we started from the assumption that the contours extracted from generated masks are closed and smooth. To enforce this assumption, we used the preprocessing method described in [77], consisting of smoothing, parametrization and resampling, in a way where the input for the fitting procedure is a uniform angular parametrization of a given contour composed of a list of points $x_{i}={\left(u\left(\theta_{i}\right),v\left(\theta_{i}\right)\right)}^{T}$ and a 1-to-1 mapping between angles $\theta_{i}$ and samples $x_{i}$ in pixel units. Then, we used a non-linear least squares minimization process for fitting an explicit model x=x(θ) based on angular parametrization:

(12) x(θ)=c+Ar(θ),

where $c={\left(c_{u},c_{v}\right)}^{T}$ is the barycenter of the ellipse, $r\left(\theta \right)={\left(\cos\theta ,\sin\theta \right)}^{T}$ is the angular unit vector, and $A=[\left(a_{uu},a_{uv}\right),\left(a_{vu},a{\;}_{vv}\right)]$ is a 2 × 2 matrix mapping the unit circle to ellipse. The proposed explicit model has various advantages. First, it depends on six parameters $Œ=\{c_{u},c_{v},a_{uu},a_{uv},a_{vu},a{\;}_{vv}\}$ all having the same dimensions (in pixels), and this makes it easy to define meaningful geometric bounds for the minimization process; second, the cost function can be computed with respect to the real geometric distance between points; finally, the Jacobian of cost function and the geometric parameters of the best-fit ellipse can be computed in closed-form. In our case, we considered as the cost function the square geometric distance weighted with the curvature computed for each point and regularized with the Tikhonov term for avoiding that the Jacobian matrix becomes singular during the minimization process:

(13) $$C\left(Œ\right)=\sum_{i}w_{i}∥x_{Œ}\left(\theta_{i}\right)-x_{i}{∥}^{2}+\tau {∥Œ∥}^{2},$$

where $w_{i}=\kappa_{i}=\frac{1}{R_{i}}=\frac{1}{∥x_{i}-c∥}$ is an estimate of the curvature of the ellipse in the point $x_{i}$ and τ is a small regularization constant (in our experiments $\tau =10^{-8}$). Hence, the fitting problem can be stated as finding the set of parameters Œ for minimizing the cost function:

(14) $${Œ}_{\mathrm{opt}}=\underset{Œ}{arg min}C\left(Œ\right),$$

which can be solved using standard methods, like the Levenberg–Marquardt (LM) [78] or Trust Regions (RTS) [79]. In our experiments, we tried both methods as implemented in the Python scipy module, without noticeable differences in the fitting accuracy. As initial values for the minimization process, we used the parameters extracted from the bounding box of the contour.

**Figure 4:**
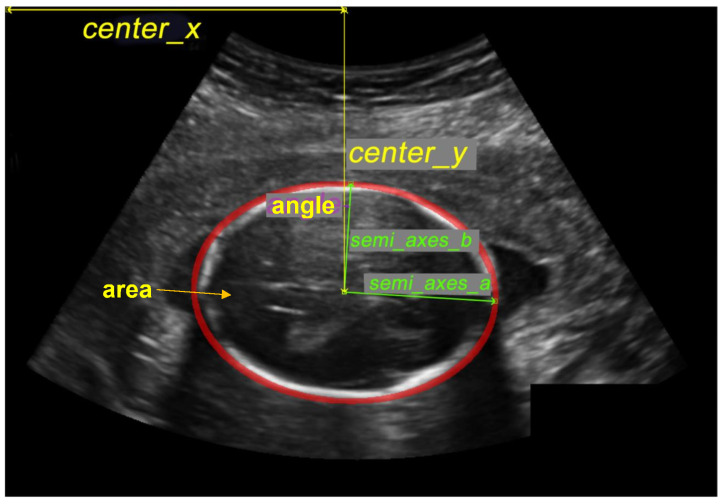
Illustration of the fetal head measurement.

Once the parametric representation of the ellipse was recovered, the geometric measurements can be computed in a closed form. Specifically, the semi-axes length and vectors can be computed by finding the extrema of the square distance between the ellipse and the center of the ellipse. According to the parametric model:

(15) $$\theta_{ext}=\underset{\theta }{arg min}{∥x\left(\theta \right)-c∥}^{2}=\underset{\theta }{arg min}{∥Ar\left(\theta \right)∥}^{2},$$

leading to the equation $A\;\dot{r}\cdot Ar=0$, with solution

(16) $$\theta_{ext}=\frac{1}{2}\arctan\frac{\left(a_{uv}^{2}+a_{vv}^{2}\right)-\left(a_{uu}^{2}+a_{vu}^{2}\right)}{2(a_{uu}a_{uv}+a_{vu}a_{vv})}+k\frac{\pi }{2}$$

from which the semi-axes vectors can be directly computed. As seen in Figure 4, the measurements of interest include:

1. center x: represents the length in millimeters between the image’s beginning pixel on the *x*-axis and the ellipse’s middle pixel.
2. center y: represents the length, in millimeters, between the image’s beginning pixel on the *y*-axis and the ellipse’s middle pixel.
3. semi-axes a: Once the ellipse’s center is determined, the semi-axes determine the radius’s maximum value based on the distance between the ellipse’s middle and its farthest point.
4. semi-axes b: Once the ellipse’s center is determined, the semi-axes determine the radius’s minimum value based on the distance between the ellipse’s middle and its nearest point.
5. angle: contains the radian value of the angle formed by the center y and the semi-axis b.
6. area: is the size of the area in millimeters that represent the fetal head.

From previous values, the equivalent diameter, biparietal diameter (BPD), occipitofrontal diameter (OFD), and HC were calculated based on the following formula:

(17) Equivalentdiameter=semiaxesa+semiaxesb

(18) BPD=semiaxesb∗2

(19) OFD=semiaxesa∗2

(20) $$HC=\pi \left(BPD+OFD\right)/2$$

To ensure that the formula that we obtained from [6] for calculating HC is more accurate than that formally used in [41], the mean difference was calculated to compare both the formulas with the HC ground truth, which was given for the whole training set. Table 3 shows that our HC measurement is the closest to the HC ground truth.

### 3.5. GA and EFW Prediction

After completing the segmentation and fetal head measurements in the previous section, eight values (features) that represent the fetal head were obtained. These values are needed to generate a new dataset for fetal GA and EFW prediction.

#### 3.5.1. Fetal Gestational Age Dataset

In the domain of fetal size and dating, Altman and Chitty [80] proposed a new formula for calculating the gestation age based on HC; later Loughna et al. [6] proved that this formula is only accurate when the fetal age is between 13 to 25 weeks. Therefore, this study used the formula recommended by Altman and Chitty [80] to label the new dataset manually, but only included GA from 13 to 25 weeks. Finally, the new dataset was used to train multi-regression models and predict GA from 10 to 40 weeks to overcome the limitation of the original formula:

$$\begin{array}{c} log_{e}\left(GA\right)=\;0.010611\times HC-0.000030321\times HC^{2}\\ +0.43498\times 10^{-7}\times HC^{3}+1.848 \end{array}$$

(21) $$GA=\exp\left(log_{e}\left(GA\right)\right)$$

Table 4 shows that we generated the new dataset from both the training and testing images. The new dataset was split into three partitions for training (13–25 weeks), validation (10–40 weeks), and testing (GA<13 and GA>25 weeks). The purpose of the validation set was to select the optimum regression model. The test set is used to compare the efficiency of the selected model, with results being obtained by an expert doctor. The mean square error (MSE) was used to evaluate different regression models, and Pearson’s r [81], to measure the statistical association between the predicted results by the regression models and the physician results based on test dataset GA prediction.

#### 3.5.2. Fetal Weight Dataset

Estimated Fetal Weight (EFW) is calculated based on Hadlock’s formula [54], which required a pre-knowledge of HC, BPD, AC, and FL. In addition, Salomon et al. [53] proposed a polynomial formula to find a new reference chart for EFW calculation which only required the knowledge of GA. This new formula (see Equation (22)) is used to estimate fetal weight in grams based on fetal GA from 20 to 36 weeks. This formula was used to label the new dataset manually, but only fetal weights for GAs between 20 to 36 weeks were used in this dataset. The new dataset was then used to train multi-regression models and predict the EFW from 10 to 40 weeks to overcome the limitations of the original formula:

(22) $$\begin{array}{c} EFW=-26256.56+4222.827\times GA-251.9597\times GA^{2}\\ +6.623713\times GA^{3}-0.0628939\times GA^{4} \end{array}$$

Table 5 shows that we generated the new dataset from both training and testing images. The new dataset was split into three partitions for training (20–36 weeks), validation (10–40 weeks), and testing (GA<20 and GA>36 weeks). The purpose of the validation set is to select the optimum regression model for fetal weight prediction. The test set is used to compare the efficiency of the selected model, with results being obtained from longitudinal reference [82]. The mean square error (MSE) was used to evaluate different regression models, and Pearson’s r [81] was used to measure the statistical association between predicted results by the regression models and longitudinal reference [82].

## 4. Experiments

In this section, the experiment set up is identified, and the three levels of evaluation for the segmentation model and the two levels of evaluation for the GA and EFW predictions are explained.

### 4.1. Training

This study’s experiments were performed on a graphics workstation, with Intel(R) Core(TM) i9-9900K CPU @ 3.60 GHz, NVIDIA GeForce RTX 2080 Ti 11 GB, and 64 G RAM. The popular Tensorflow 2.6.0 and Keras 2.4.0 were chosen for the deep learning framework. All segmentation models were trained using the same hyperparameter settings as seen in Table 2; each model was trained for 100 epochs, and the training time was reported. The input size of model training for the first experiment was 64 × 64, and the second was 128 × 128.

### 4.2. Segmentation Models Evaluation

Three levels of evaluation were conducted to quantitatively analyze and evaluate the segmentation model’s performance, as seen in Table 6.

#### 4.2.1. Level 1: Segmentation Evaluation

Eight indices (Equations (23)–(30)) were used to evaluate segmentation model performance. These indices included area under the curve (AUC), accuracy (ACC), mean intersection over union (mIoU), precision (Pre), recall, dice similarity coefficient (DSC), mean squared error (MSE), and mean pixel accuracy (mPA), as defined below:

$$\begin{array}{c} True\;Positive\;Rate\left(TPR\right)=\frac{True\;Positive(TP)}{True\;Positive(TP)+False\;Negative(FN)}\\ False\;Positive\;Rate\left(FPR\right)=\frac{False\;Positive(FP)}{False\;Positive(FP)+True\;Negative(TN)} \end{array}$$

(23) AUC=TPR−FPR

(24) $$Accuracy=\frac{TP+TN}{TP+TN+FP+FN}$$

(25) $$mIoU\left(U,V\right)=\sum_{i=1}^{C}\frac{\left|U\cap V\right|}{\left|U\cup V\right|}=\sum_{i=1}^{C}\frac{TP}{TP+FP+FN}$$

(26) $$Pre=\frac{TP}{TP+FP}$$

(27) $$Recall=\frac{TP}{TP+FN}$$

(28) $$DSC=\frac{2*Pre*Recall}{Pre+Recall}=\frac{2*TP}{2*TP+FP+FN}$$

(29) $$MSE=\sum_{i=1}^{C}{\left(G_{i}-P_{i}\right)}^{2}$$

(30) $$mPA=\frac{\sum_{i=1}^{C}TP}{\sum_{i=1}^{C}TP+FP}$$

#### 4.2.2. Level 2: Post-Processing Evaluation

This study compared predicted masks using different models with ground truth masks to evaluate the predicted mask in terms of quality assessment and smoothing (post-processing). For this purpose, five indices [83] (Equations (31)–(35)) were used, including mean Hausdorff distance (mHD), mean surface distance (MSD), relative volume difference (RVD), mean structural similarity index (MSSIM), and peak signal-to-noise ratio (PSNR):

(31) $$\begin{array}{c} mHD\left(P,G\right)=\frac{\frac{1}{P}\sum_{p\in P}max\;d\left(p,g\right)+\;\frac{1}{G}\sum_{g\in G}max\;d\left(p,g\right)}{2} \end{array}$$

(32) $$MSD=\frac{1}{2}\left[\hat{d}\left(S_{p},S_{g}\right)+\hat{d}\left(S_{g},S_{p}\right)\right]$$

(33) $$RVD\left(P,G\right)=\frac{\left|G\right|-\left|P\right|}{\left|P\right|}$$

(34) $$MSSIM\left(G,P\right)=\frac{1}{M}\sum_{j=1}^{M}SSIM\left(g_{j},p_{j}\right)$$

(35) $$PSNR\left(P,G\right)=10\;log\left(\frac{255^{2}}{MSE(P,G)}\right)$$

#### 4.2.3. Level 3: Measurement Evaluation

To ensure the set of values obtained through the measurement algorithm, three indices (Equations (28), (31) and (36)) were used to evaluate the test dataset, including mHD, DCS, and mean absolute difference (MAD), as defined below:

(36) $$MAD=\frac{\sum \left|HC_{p}-HC_{g}\right|}{n}$$

### 4.3. Evaluation of GA and EFW Prediction

Regression models were used for the estimated fetal GA and EFW predictions. MSE (Equation (29)) was used to evaluate and select the best regression model. Pearson’s r [81] (Equation (37)) was used to evaluate the predicted value (GA and EFW) by calculating the statistical association between our model, the medical doctor, and the longitudinal reference.

(37) $$r=\frac{\sum_{i=1}^{n}\left(x_{i}-\bar{x}\right)\left(y_{i}-\bar{y}\right)}{\sqrt{\sum_{i=1}^{n}{\left(x_{i}-\bar{x}\right)}^{2}{\left(y_{i}-\bar{y}\right)}^{2}}}$$

where *r* is correlation coefficient, $x_{i}$ is the values of the *x*-variable in a sample, $\bar{x}$ is the mean of the values of the *x*-variable, $y_{i}$ is the values of the *y*-variable in a sample, $\bar{y}$ is the mean of the values of the *y*-variable.

## 5. Results and Discussion

The first part of this section presents the obtained results for the different models’ segmentation efficiency, mask quality assessment (post-processing), measurement performance, and a comparison with the previous state-of-the-art. The second part presents the obtained result for the fetal GA and EFW regression models’ efficiency and clinical validation.

### 5.1. Segmentation Performance

**Figure 5:**
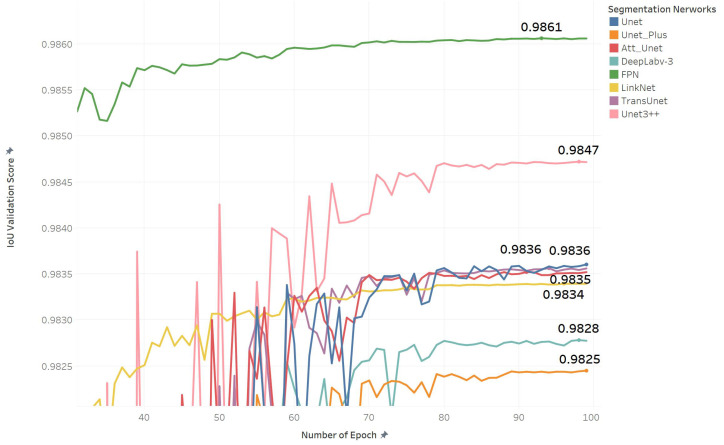
Segmentation networks performance based on IoU validation score during training with 128 × 128 input size.

Figure 5 shows that all models obtained a validation score above 0.98 IoU during training. The FPN reached 0.9861 IoU, which is slightly better than other models. It is a 0.04 IoU improvement, compared to the lower performing model LinkNet, which has a 0.9825 IoU. UNet3+ obtained the second-best value but took a long time to train, as seen in Table 7. Therefore, UNet3+ was excluded from the weighted voting algorithm. LinkNet, Deeplab3,TransUNet, and UNet Plus obtained low scores of 0.982, 0982, and 0.983, respectively; therefore, they were excluded during the weighted voting algorithm. The FPN, UNet, and AttUNet models obtained the highest IoU score with a low training time. These models were used to perform weighted voting and to select the optimum weight for our ETLM. Table 7 reports eight indices that are used to evaluate each model’s segmentation performance [84].

The overall result proves that transfer learning using EfficientNetB0 achieved promising results, despite a low input size and less training time. Therefore, this study proves that transfer learning can develop a lightweight model, which was a challenge for medical image segmentation tasks. With an input size of 128 × 128 and no augmentation, results may vary from one model to another. It can be seen from the two indices, mIoU and MSE, that the FPN and AttUNet achieved the best result with the average training time. Further, with input size 64 × 64 and augmentation, ETLM outperformed all other models in terms of ACC, mIoU, Pre, Recall, DSC, AUC, and mPA. In the case of input size 128 × 128 with augmentation, ETLM outperformed all other models in terms of ACC, mIoU, Pre, Recall, AUC, MSE, and mPA. Finally, as seen in Table 7, all indices reported during validation showed that ensemble learning could add slight improvements to the segmentation model and predict image masks. However, these new predicted masks had to be post-processed for edge smoothing and required quality assessment tests, as discussed in the following subsection.

### 5.2. Measurements Performance

#### 5.2.1. Post-Processing Evaluation

**Figure 6:**
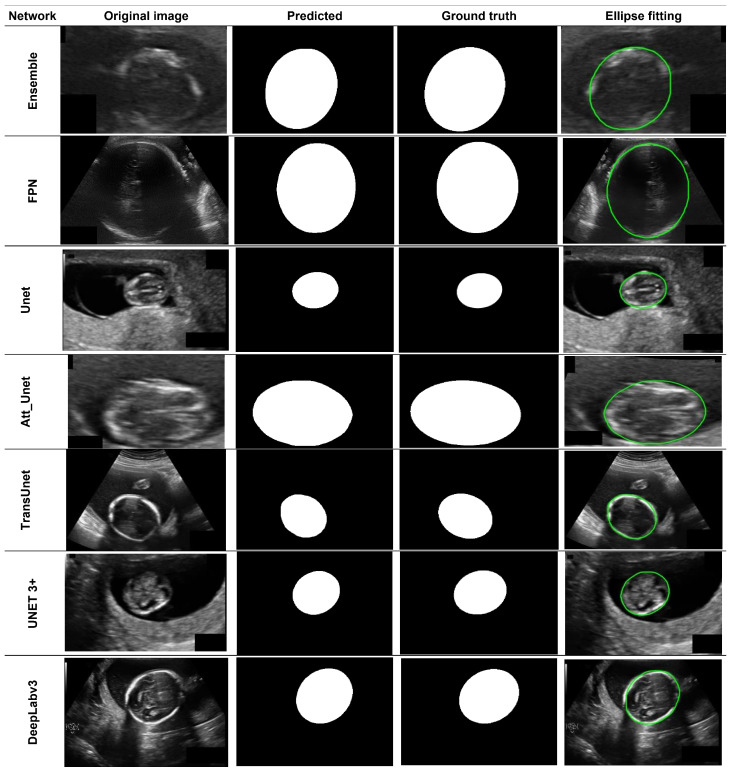
Qualitative comparison of segmentation performance of networks on a fetal head ultrasound image. The predicted mask, ground truth, and original image boundaries are shown. The predicted masks using different networks and the proposed ETLM are shown in the first row.

Figure 6 presents samples of original images, predicted masks after post-processing, ground truths, and ellipse fitted masks; however, it is challenging to identify differences and similarities by looking at the predicted masks and ground truths. Therefore, this study performed a mask quality assessment test shown in Table 8, to prove that the promised result obtained during the level one evaluation is realistic and reliable.

Table 8 shows a comparison between two distinct groups of predicted masks; the first group was predicted using various segmentation networks trained with a 64 × 64 input size. The other group used networks that trained with a 128 × 128 input size. In both cases, the results indicate that ETLM is more like the ground truth mask, where minimum mHD, MSD, RVD, and maximum MSSIM and PSNR were obtained using masks predicted by ETLM with post-processing. However, some results may vary slightly, as seen in the case of the 128 × 128 FPN, which obtained minimum mHD, but the ETLM performance was best in other indices. The RVD is always negative, as seen in Table 8, which means that in all cases, the predicted mask size (fetal head contour) was bigger than the ground truth in the masks predicted by different networks. However, ETLM minimized this difference to 0.0016 to achive the best similarity with the ground truth. Overall, the level two evaluation proved that the predicted masks obtained by this study’s ETLM are remarkably close to the ground truth, with a difference of 0.011, as reported by MSSIM (see Figure 6).

#### 5.2.2. Fetal Head Measurement Evaluation

Fetal head measurements were evaluated on the testing dataset, which consisted of 355 images. Unfortunately, the ground truth for this dataset is not available to the public; therefore, the measurement evaluation result was obtained by submitting measurement values to the dataset website (https://hc18.grand-challenge.org/ 11 August 2022) and obtaining the mHD, MAD, and DCS, as shown in Table 9.

### 5.3. Comparative Analysis

Table 10 provides a comprehensive comparison between our ETLM and the published results reported in the literature. First, the ETLM outperformed the state-of-the-art models in the segmentation task regarding ACC, mIoU, Pre, and mPA. Second, the results of this study are better than [32,36,39,43,47], in terms of MAD, and better than [32,39,42] in terms of mHD. However, the result in this study is inferior to the results found in [41,49] because the models used in those studies were heavy and trained for more than 30 h with high input resolution, making the models very expensive in terms of required resources and time. Finally, a model weight comparison showed that the lightweight ETLM used in this study is superior, because promising results with very low resolution (128 × 128) and less training time (2 h) were achieved. This study proves that ensemble and transfer learning overcomes medical image segmentation challenges such as low image intensity, the need for expensive resources, long training time, and heavy model deployment.

### 5.4. GA and EFW Prediction Performance

For fetal GA and EFW prediction, we trained 17 regression models on each dataset independently. Because the dataset contains large numerical values, a log transformation was applied to both datasets before training, making the highly skewed distributions less skewed. The performance of each model was evaluated using MSE, and the result was reported in Table 11. This task aimed to address the limitation of both formulas (see Equations (21) and (22)) used to estimate the GA and EFW. Therefore, the regression model was used to predict GA for the fetus when the GA of the fetus was 13>GA>25, and the EFW for the fetus when the GA of the fetus was 20>GA>36. In both cases, the ground truth was non-existent because both formulas had limitations, and a GA and EFW could not be calculated in the mentioned periods; therefore, the following steps were taken:

1. Validation of predicted GA: 50 random samples images taken from the testing set (13>GA>25) were given to a senior attending physician with 21 years of experience in maternal-fetal medicine, to estimate GA. We used Pearson’s r to measure the strength of a linear association between the physician prediction and the model prediction for the same sample set. Because we do not have any pre-knowledge of the dataset in terms of ethnicity or location, the GA may vary based on these factors; therefore, in this work, we tried to predict the GA in the 50th percentile, and considered the median.
2. Validation of predicted EFW: In the case of EFW, the senior physician could not estimate the EFW based on fetal head images and required more factors such as FL, AC, and CRL. Therefore, a growth chart taken from a longitudinal reference was used for estimated fetal weight, regardless of fetal sex [82]. Then, Pearson’s r was used to measure the strength of the linear association between the longitudinal reference and the model prediction for the same sample set that fell in the range of 20>GA>36. This study tried to predict the EFW in the 50th percentile and considered the median for the above mentioned reason.

Table 11, shows that most regression models achieved a promising result in GA and EFW datasets based on MSE. In the GA validation dataset, polynomial regression and Deep NN achieved a lower MSE of 0.0003 and 0.00072, respectively. However, to ensure the reliability of each model, all models were used to predict the 50th percentile of GA. The predicted GA was then compared with the physician’s estimations using Pearson’s r. After comparing the predicted GA with the physician’s estimation, Table 11 shows that Deep NN and polynomial regression outperformed all regression models for predicting the GA, with Pearson’s r of 0.9978 and 0.9958, respectively.

For Fetal EFW, LinearSVR, XGBRFRegressor, and linear regression achieved the lower MSE in the EFW validation dataset, as reported in Table 11. Nonetheless, all the models were used to predict the 50th percentile of EFW in the test dataset to ensure the reliability of each model’s prediction. Then, it was compared with the longitudinal reference table, as seen in Appendix A Table A1. As a result, Pearson’s r showed that LinearSVR outperformed all the models and predicted the EFW in the 50th percentile with the highest association with the longitudinal reference (r = 0.9989). In addition, XGBRFRegressor showed a low MSE during validation, and a low association with the longitudinal reference.

Overall, most regression models could predict the GA and EFW in the 50th percentile, as seen from Pearson’s results in Table 11. It is concluded that the regression models in this study address the limitations of the formulas currently used to calculate GA and EFW in the specific period. Without limitation, these models only required measurement of the fetal head to calculate GA and EFW from the 10th week to the 40th week. This study is the first work that utilized machine learning to predict the GA and EFW based on fetal head images. A sample of model prediction for GA and EFW was added to (Supplementary File S1 and File S2), respectively.

## 6. Strength and Limitations

Including US machines in various medical settings is advised; however, this is not always feasible, due to the cost of purchasing multiple devices or portability concerns. Mobile Health companies such as Clarius (Clarius Mobile Health Corp., Vancouver, BC, Canada) [85] developed portable pocket handheld ultrasound scanners that represent a promising tool in regional anesthesia procedures and vascular accesses [86]. Furthermore, these portable devices are still examined for extensive imaging, such as prenatal scans, which require a lightweight AI system to maintain high accuracy and low resource. Therefore, in this work, we deployed lightweight architectures that can be used in portable devices without client-server communication. The architectures resulted in fast training on low-end machines and fast inference without the need for complex client-server architecture that would pose issues for data privacy and security limitations related to image resolution that can affect measurement accuracy. In addition to fetal head segmentation, a regression model was employed to predict GA and EFW in the 50th percentile in all trimesters based on fetal head features, which current methods cannot do. Furthermore, the framework in this study can be extended to build a fully automatic AI system in the client-server to provide a detailed report for any fetal head ultrasound images.

Despite the study’s strengths, the framework still has some constraints that will need to be overcome in the future. First, downsampling the original images reduced the measurement accuracy. For example, subsampling images from 128 × 128 to 64 × 64 reduced the PSNR value by 3.1 and mHD by 0.17 mm, as seen in Table 8. Second, fetal GA and EFW may vary slightly from one group to another, based on ethnicity and gender. This study did not have this information, so the 50th percentile was predicted as the median. Moreover, the clinical appliance has to be decided by medical personnel, since the existing differences between the actual image and the one generated by the proposed model could be substantial in the medical field.

## 7. Conclusions and Future Work

This work proposed a new pipeline that utilized transfer learning and ensemble learning to build ensemble models called ETLM. Eight segmentation networks were evaluated to build an ensemble model based on the weighted voting method for fetal head segmentation. These segmented masks were used to accurately measure HC, BPD, OFD, and other values in ultrasound images. Masks segmented by each model went through a quality assessment test to ensure the efficiency of ETLM, and were compared with other independent models. Our experimental results show that the proposed pipeline achieved comparable performance to state-of-the-art models in segmentation and measurement. Further, regression models showed that of the features obtained from the segmented fetal images to build a new dataset for GA and EFW, only fetal head images were required to predict GA and EFW. The results of this study were validated with the assistance of an expert physician and longitudinal reference. This study is the first work that provides a completed approach from image segmentation to GA and EFW prediction. Future work will include a full adoption of transfer learning based on a model trained on ultrasound images, regardless of the domain of the images. Further, a traditional machine learning classifier will be used to find the best features to reduce ultrasound images’ intensity and noise. Finally, the cavum septum pellucidum and the lateral ventricle will be segmented, measured, and compared with the ultrasound machine.

Future work will include a full adoption of transfer learning based on a model trained on ultrasound images, regardless of the domain of the images. Further, a traditional machine learning classifier will be used to find the best features that will reduce the intensity and the noise in the ultrasound images. Finally, we will segment and measure the cavum septum pellucidum and the lateral ventricle, and compare our results with the ultrasound machine.

## Figures and Tables

**Table 1:** Distribution of dataset during the various trimesters of pregnancy.

Trimesters of Pregnancy	Training Sets	Testing Sets
First trimester	165	55
Second trimester	693	233
Third trimester	141	47
Total	999	335

**Table 2:** The selected segmentation models and their details.

Model Name	Backbone	Output Function	Normalization	One-Hot Encoding	Optimizer	Loss Function	Batch Size	Epoch	Input Size	Trainable Params
UNet	EfficientNetB0	Softmax	0 to 1	0 = black pixel 1 = weight pixel	RMSprop + Scheduler Learning Rate Step Decay	Categorical Focal Jaccard loss	32	100	64 × 64 128 × 128	2,776,114
UNet_plus										2,389,042
Att_UNet										2,614,725
UNet 3+										3,183,330
TransUNet										2,218,322
FPN										4,911,614
LinkNet										6,049,342
DeepLabv3										4,027,810

**Table 3:** Comparing two HC measurement formulas with the HC ground truth using the mean difference.

Formula	Mean HC of the GT	Mean HC by Each Formula	Mean Difference
Our formula	174.3831 mm	174.2411 mm	**−0.14203**
Other Formula		178.3705 mm	3.9874

**Table 4:** Fetal gestational age dataset.

	GA Validation	GA Training	GA Testing
Dataset	(10–40) weeks	(13–25) weeks	GA < 13 GA > 25
Training	999	692	307
Testing	335	232	103
Total	1334	924	410

**Table 5:** Estimated fetal weight dataset.

	EFW Validation	EFW Training	EFW Testing
Dataset	(10–40) weeks	(20–36) weeks	GA < 20 GA > 36
Training	999	551	448
Testing	335	175	160
Total	1334	726	608

**Table 6:** Evaluation levels for segmentation model.

	Level 1 Segmentation Evaluation	Level 2 Post-Processing Evaluation	Level 3 Measurement Evaluation
Total	Training 80% Validation 20%	Validation 100%	Validation 100%
Augmented	7992 1998		
Training Set		999	
Testing Set			335

**Table 7:** Level one: performance evaluation and comparison of segmentation results for all models with various input sizes, and with and without augmentation.

Model Trained with Input Size	Network	Augmentation	ACC	mIoU	Pre	Recall	DSC	AUC	MSE	mPA	Time (min)
**128 × 128**	UNet	No	0.9855	0.9667	0.9780	0.9740	0.9854	0.9820	0.014	0.9711	0:11:02
	UNet_plus		0.9852	0.9662	0.9710	**0.9815**	0.9851	0.9842	0.014	0.9665	0:10:43
	Att_UNet		0.9862	0.9680	0.9769	0.9787	**0.9862**	0.9841	**0.013**	0.9721	0:11:35
	UNet 3+		0.9856	0.9671	0.9770	0.9766	**0.9856**	0.9830	0.014	0.9693	0:25:20
	TransUNet		0.9852	0.9662	0.9783	0.974	0.9852	0.9821	0.014	0.9756	0:12:08
	FPN		**0.9866**	**0.9693**	0.9790	0.9778	0.9860	0.9840	**0.013**	0.9730	0:13:29
	LinkNet		0.9857	0.9673	0.9770	0.9760	0.9856	0.9830	0.014	0.9692	0:12:14
	Deeplabv3		0.9852	0.9660	**0.9791**	0.9727	0.9845	0.9817	0.014	**0.9763**	0:11:04
**64 × 64**	UNet	Yes	0.9917	0.9810	0.9870	0.9859	0.9916	0.9900	0.008	0.9870	0:39:00
	UNet_plus		0.9898	0.9767	0.9843	0.9833	0.9896	0.9880	0.010	0.9815	0:38:18
	Att_UNet		0.9919	0.9815	0.9881	0.9863	0.9919	0.9900	0.008	0.9875	0:40:38
	UNet 3+		0.9920	0.9816	0.9883	0.9862	0.9919	0.9904	0.007	0.9892	1:16:44
	TransUNet		0.9913	0.9802	0.9873	0.9851	0.9912	0.9896	0.008	0.9873	0:44:44
	FPN		0.9926	0.9831	0.9887	0.9878	0.9925	0.9913	**0.007**	0.9886	0:48:51
	LinkNet		0.9912	0.9800	0.9868	0.9854	0.9911	0.9896	0.008	0.9860	0:46:13
	Deeplabv3		0.9908	0.9790	0.9869	0.9838	0.9903	0.9889	0.009	0.9842	1:07:17
	ETLM		**0.9928**	**0.9841**	**0.9892**	**0.9881**	0.9934	**0.9918**	0.008	**0.9904**	NA
**128 × 128**	UNet	Yes	0.9928	0.9820	0.9888	0.9886	0.9928	0.9917	0.007	0.9898	0:37:15
	UNet_plus		0.9923	0.9807	0.9879	0.9879	0.9922	0.9911	0.007	0.9877	0:35:10
	Att_UNet		0.9928	0.9819	0.9887	0.9885	0.9927	0.9916	0.007	0.9891	0:38:59
	UNet 3+		0.9933	0.9832	0.9900	0.9890	**0.9933**	0.9921	0.006	0.9908	1:40:12
	TransUNet		0.9928	0.9819	0.9890	0.9884	0.9927	0.9916	0.007	0.9892	0:38:29
	FPN		0.9939	0.9846	0.9908	0.9899	0.9938	0.9928	0.006	0.9905	0:42:47
	LinkNet		0.9927	0.9817	0.9892	0.9879	0.9926	0.9914	0.007	0.9886	0:36:30
	Deeplabv3		0.9926	0.9828	0.9886	0.9878	0.9923	0.9913	0.007	0.9884	0:43:11
	ETLM		**0.9942**	**0.9853**	**0.9913**	**0.9903**	0.9908	**0.99316**	**0.005**	**0.9914**	NA

**Table 8:** Level two: predicted mask (post-processed) quality assessment for models with various input sizes.

Model Trained with Input Size	Network	Original Training Images	mHD (mm)	MSD (mm)	RVD	MSSIM	PSNR
**64 × 64**	ETLM	Yes	**0.927634**	**0.0034989**	**−0.00387**	**0.98108–0.98255**	**25.142206**
	FPN		1.186636	0.0049680	−0.01237	0.97322–0.97544	23.47897
	UNet		1.118771	0.0048532	−0.01213	0.97352–0.9757270	23.5358
	Att_UNet		1.512662	0.0049149	−0.01222	0.973301–0.9755263	23.505971
	Trans_UNet		1.118771	0.0049047	−0.01208	0.97344304–0.97563850	23.50993
**128 × 128**	ETLM	Yes	0.753095	**0.0018117**	**0.001639**	**0.989922–0.990706**	**28.247806**
	FPN		**0.625412**	0.0020034	−0.00264	0.9888480–0.9896689	27.536022
	UNet		1.250824	0.0020566	−0.00196	0.98856–0.989421	27.484995
	Att_UNet		0.988862	0.0020950	−0.00177	0.988375–0.989247	27.41142
	Trans_UNet		0.753095	0.0020579	−0.00243	0.988523–0.98937365	27.43699

**Table 9:** Level three: measurement evaluation based on testing dataset.

Model Trained with Input Size	Network	Original Testing Images	mHD (mm)	MAD (mm)	DCS
128 × 128	ETLM	Yes	1.6715	1.8735	0.9716

**Table 10:** Comprehensive comparison with state-of-the-art models.

Type of Comparison	Segmentation				Measurement			Model Weight				
**Network**	**ACC**	**mIoU**	**Pre**	**mPA**	**DSC**	**MAD**	**mHD**	**Model Trained ** **with Input Size**	**Bach ** **Size**	**GPU RAM**	**Epochs**	**Training Time**
ETLM [UNet: Att_UNet: FPN] [0.3: 0.3: 0.4]	**0.9942**	**0.9853**	**0.9913**	**0.9914**	0.9716	1.87	1.67	**128 × 128**	**32**	**11 GB**	**300**	**2:01 h**
VNet-c [43]	0.9888	0.9594	0.9767	NA	0.9791	1.89	NA	512 × 512	4	6 GB	300	53:35 h
VSAL [42]	NA	NA	N/A	0.990	0.9710	NA	3.234	256 × 256	4	24 GB	100	17:30 h
SAPNet [49]	NA	0.9646	NA	0.9802	0.9790	1.81	**1.22**	480 × 320	10	11 GB	700	NA
Regression CNN [47]	NA	NA	NA	NA	0.9776	1.90	1.32	800 × 800	16	NA	1500	NA
DAG V-Net [41]	NA	NA	NA	NA	**0.9793**	**1.77**	1.27	768 × 512	2	11 GB	20	30 h
MTLN [32]	NA	NA	NA	NA	0.9684	2.12	1.72	800 × 540	NA	11 GB	200	15 h
UNet [36]	NA	NA	NA	NA	0.9731	2.69	NA	216 × 320	4	32 GB	100	NA
DSCNN [40]	NA	NA	NA	NA	0.9689	NA	NA	NA	NA	NA	NA	NA
MS-LinkNet [39]	NA	NA	NA	NA	0.9375	2.27	3.70	NA	10	11 GB	150	18 h

**Table 11:** Result and validation of multiple regression models for GA and EFW prediction.

	Fetal GA Prediction in the 50th Percentile (13 > GA > 25) Week		EFW Prediction in the 50th Percentile (20 > GA > 36) Week	
Regression model	MSE	Pearson’s r	MSE	Pearson’s r
Polynomial Regression	**0.00033**	**0.9958**	9.08723	0.9422
Linear Regression	0.00205	0.9899	**0.00035**	**0.9988**
Random Forest Regressor	0.00842	0.9511	6.54380	0.9844
XGBRFRegressor	0.02268	0.9505	**0.00018**	0.9847
Neural network	0.01392	0.9805	0.00256	0.9946
KNeighbors Regressor	0.00921	0.9582	0.00214	0.9841
SGDRegressor	0.00219	0.9901	0.00146	0.9968
AdaBoostRegressor	0.01086	0.9505	0.00100	0.9843
BaggingRegressor	0.01081	0.9832	0.00281	0.9964
StackingRegressor	0.00824	0.9506	6.93890	0.9843
LinearSVR	0.00199	0.9901	**0.00054**	**0.9989**
LGBMRegressor	0.01011	0.9514	7.72867	0.9843
Lasso	0.08300	NA	0.17339	0.8507
VotingRegressor	0.00248	0.9909	0.00031	0.8507
BayesianRidge	0.00206	0.9899	0.00035	0.9988
Deep NN	**0.00072**	**0.9978**	0.00068	NA

## Data Availability

Data and code are available upon request.

## Supplementary Materials

The following supporting information can be downloaded at: https://www.mdpi.com/article/10.3390/diagnostics12092229/s1, File S1: Sample of Models Prediction for GA against Doctor calculation. File S2: Sample of Models Prediction for EFW against Longitudinal reference in 50th Percentile.

## Appendix A. Longitudinal Reference

**Table A1:** Growth chart for estimated fetal weight regardless of fetal sex.

Gestational Age (Weeks)		Estimated Fetal Weight (g) by Percentile								
		2.5	5	10	25	50	75	90	95	97.5
	14	70	73	78	83	90	98	104	109	113
	15	89	93	99	106	114	124	132	138	144
	16	113	117	124	133	144	155	166	174	181
	17	141	146	155	166	179	193	207	217	225
	18	174	181	192	206	222	239	255	268	278
	19	214	223	235	252	272	292	313	328	340
	20	260	271	286	307	330	355	380	399	413
	21	314	327	345	370	398	428	458	481	497
	22	375	392	412	443	476	512	548	575	595
	23	445	465	489	525	565	608	650	682	705
	24	523	548	576	618	665	715	765	803	830
	25	611	641	673	723	778	836	894	938	970
	26	707	743	780	838	902	971	1038	1087	1125
	27	813	855	898	964	1039	1118	1196	1251	1295
	28	929	977	1026	1102	1189	1279	1368	1429	1481
	29	1053	1108	1165	1251	1350	1453	1554	1622	1682
	30	1185	1247	1313	1410	1523	1640	1753	1828	1897
	31	1326	1394	1470	1579	1707	1838	1964	2046	2126
	32	1473	1548	1635	1757	1901	2047	2187	2276	2367
	33	1626	1708	1807	1942	2103	2266	2419	2516	2619
	34	1785	1872	1985	2134	2312	2492	2659	2764	2880
	35	1948	2038	2167	2330	2527	2723	2904	3018	3148
	36	2113	2205	2352	2531	2745	2959	3153	3277	3422
	37	2280	2372	2537	2733	2966	3195	3403	3538	3697
	38	2446	2536	2723	2935	3186	3432	3652	3799	3973
	39	2612	2696	2905	3135	3403	3664	3897	4058	4247
	40	2775	2849	3084	3333	3617	3892	4135	4312	4515
